# A Simple Trick to Increase the Areal Specific Capacity of Polypyrrole Membrane: The Superposition Effect of Methyl Orange and Acid Treatment

**DOI:** 10.3390/polym14214693

**Published:** 2022-11-03

**Authors:** Zahra Roohi, Frej Mighri, Ze Zhang

**Affiliations:** 1 Department of Chemical Engineering, Faculty of Sciences and Engineering, Université Laval, Quebec, QC G1V 0A6, Canada; 2 Biomaterials Group, Division of Regenerative Medicine, Research Center of CHU de Québec—Université Laval, Quebec, QC G1L 3L5, Canada; 3 Department of Surgery, Faculty of Medicine, Université Laval, Quebec, QC G1V 0A6, Canada

**Keywords:** polypyrrole, methyl orange, sulfuric acid, interfacial polymerization, energy storage, supercapacitor

## Abstract

Polypyrrole (PPy) is one of the attractive conducting polymers that have been investigated as energy storage materials in devices like supercapacitors. Previously, we have reported a free-standing soft PPy membrane synthesized through interfacial polymerization in which methyl orange (MO) and ferric chloride were used as nano template and oxidant. In this work, we report that the presence of MO and the treatment of the PPy–MO membrane with sulfuric acid can dramatically increase the specific capacitance of the membrane. The properties of the membranes were evaluated using scanning electron microscope (SEM) for morphology, Fourier transform infrared spectroscopy (FTIR) and X-ray photoelectron spectroscopy (XPS) for chemistry, thermogravimetric analysis (TGA) for thermal stability, and cyclic voltammetry (CV), and electrochemical impedance spectroscopy (EIS) for electrochemical activity. It was found that the areal specific capacitance of the PPy membrane increased from 2226 mF/cm^2^ to 6417 mF/cm^2^ and the charge transfer resistivity decreased from about 17 Ω to 3 Ω between 10,000 and 0.1 Hz due to the presence of MO and the acid treatment. It is likely that the superposition effect of MO and acid treatment helped the charge transfer process and consequently enhanced the charge storage performance and specific capacitance of the PPy membrane.

## 1. Introduction

Conducting polymers have attracted much scientific attention due to their unique properties, such as electrical conductivity and electroactivity. While they show high electrical conductivity, their mechanical properties are generally poor compared to other types of polymers [1]. However, the preparation method, such as chemical or electrochemical polymerization, can have a remarkable effect on polymer structure, final polymer form and properties. For example, the nanostructure of conducting polymers has a significant impact on their properties, such as electrical conductivity and specific capacitance [2]. Indeed, nanostructured conducting polymers have been used in new technologies as wearable energy storage devices [2]. Among conducting polymers, polypyrrole (PPy) has been widely studied due to its unique properties, such as the high electrical conductivity, ease of synthesis, low cost, and reversible redox reactions [2,3,4]. In particular, the electrical conductivity and the reversible redox activity give PPy a significant potential as an energy storage material in supercapacitors. Based on the charge storage mechanism, there are two main groups of supercapacitors, i.e., electrical double-layer capacitors (EDLCs) and pseudocapacitors. Usually, pseudocapacitors show higher electrical capacitance and energy density than EDLCs [5]. Due to its redox property, PPy has been extensively investigated to increase its pseudocapacitance. Mini et al. [6] reported a cone-shaped nanostructure of PPy modified with ruthenium dioxide (RuO_2_), which showed a specific capacitance of ~151 mF/cm^2^ at a scan rate of 10 mV/s. In another approach, Zhang et al. [7] prepared a flexible composite membrane using reduced graphene oxide and PPy nanowires, showing a specific capacitance of 175 mF/cm^2^ at 10 mV/s in 2 M KCl aqueous solution. Wang et al. [8] also succeeded in developing a tungsten trioxide (WO3)@PPy core–shell nanowire electrode, which exhibited a specific capacitance of 253 mF/cm^2^ at 10 mV/s. Supercapacitors based on PPy, hydrogel, graphene or graphene oxide, and transition metals such as manganese oxide showed the specific capacitance of 210.7 mF/cm^2^, 545 F/g, and 786.6 F/g, as reported in a recent review [9]. A composite of PPy–sodium dodecyl sulphate (SDS)-polyvinyl alcohol (PVA) has presented a specific capacitance of 950 mF/cm^2^ at current density of 1.6 mA/cm^2^ [10]. A specific capacitance of 203 mF/cm^2^ at current density of 1 mA/cm^2^ was reported for the PPy/l-Ti_3_C_2_ films, in which the PPy film was sandwiched between Ti_3_C_2_ layers [11]. In another work, a nanocomposite made of CNO/SDS/PPy or a bilayer of CNO/PPy was reported, showing a specific capacitance of 800 and 1300 F/g, respectively [10].

Based on many works, PPy is considered a good choice for energy storage devices such as supercapacitors due to its electrochemical and electrical properties, reversible redox performance, ease of synthesis, low cost, and environmental stability. In addition, the working range of PPy is wider than polyaniline (PANI), and it can work not only in an acidic environment, but also in a neutral electrolyte [12]. These characteristics make PPy a very suitable candidate to be used in pseudocapacitors.

We have previously reported a truly flexible PPy membrane without compounding with any other material [13]. This membrane has micro/nano structures and is very light and soft, making it an ideal candidate for supercapacitors. Recently, Zhang et al. reported that this membrane had a specific capacitance as high as 509.8 F/g at current density of 0.5 A/g [14].

In this work, the role of methyl orange (MO) and acid treatment was investigatedIt was found that MO not only imposed a nanostructure with a high surface area to the membrane, but also improved the electrical properties of the membrane. The superpositionary effect of MO and acid treatment dramatically increased the electrical and electrochemical performance of the PPy membrane, resulting in an unusually high areal specific capacitance.

## 2. Materials and Methods

### 2.1. Materials

Pyrrole (Py, ≥98%, Fisher Scientific, Ottawa, ON, Canada) was distilled and stored at 4 °C before being used for polymerization. Ferric chloride hexahydrate (FeCl_3_·6H_2_O, 98%, Sigma-Aldrich, Milwaukee, WI, USA) as oxidant, MO (ACS reagent, dye content 85%, Sigma-Aldrich, St. Louis, MO, USA) as nano-scale template, and sulfuric acid (H_2_SO_4_, 95–98%, MAT, Quebec, QC, Canada) were all analytical grade and used as received.

### 2.2. Preparation and Acid Treatment of PPy Membrane

To prepare the soft PPy membrane [9], 18.2 g of FeCl_3_·6H_2_O and 0.5 g of MO were added to 70 mL and 250 mL of deionized water, respectively. Both solutions were put in an ultrasonic bath in order to dissolve the materials completely. Then, the MO solution was placed on a magnetic stirring plate, and the ferric chloride solution was added slowly under vigorous stirring for 30 min. A volume of 3 mL of the freshly distilled pyrrole monomer was added to 150 mL of chloroform in a beaker, followed by a slow injection of the ferric chloride/MO solution on top of the pyrrole/chloroform solution. The injection process must be slow to avoid any significant agitation at the water/chloroform interface. The beaker was then covered with an aluminum foil to avoid light exposure and was kept at 4 °C for 48 h. The membranes harvested before this time are too thin and weak; and after 2 days, the membrane starts to become thicker but fragile. The membrane formed at the interface of the aqueous and the organic phases was gently collected, and named PPy–MO membrane. The membrane without MO, named PPy membrane, was obtained after repeatedly washing the membrane in a solution made of ethanol, HCl (37%), and deionized water (69:14:17 %*v*/*v*). The thickness of the membranes was about 0.1 mm.

**Figure 1:**
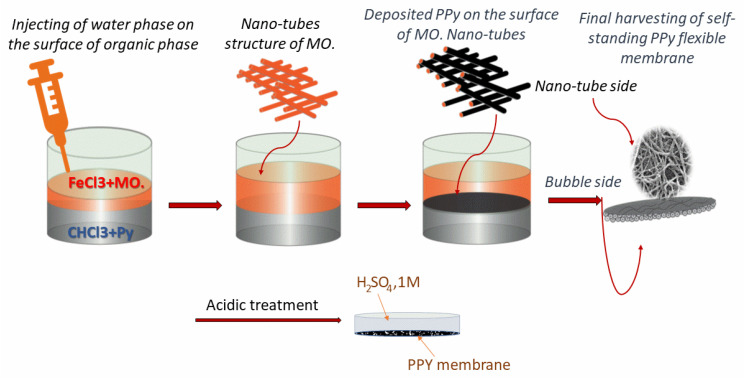
Illustration of how the flexible PPy membrane is prepared.

Acid treatment (AT) was performed by immersing the as-synthesized PPy–MO membranes in a solution of 1M sulfuric acid for 2 h. For the PPy membrane, this step was performed after the washing process. The main steps of membrane preparation are showed in Figure 1. The acid treated PPy and PPy–MO membranes were named PPy–AT and PPy–MO–AT, respectively.

## 3. Characterizations

### 3.1. Electrical and Electrochemical Characterizations

#### 3.1.1. Resistivity Measurement

The sheet resistivity (Ohm·cm) was measured at room temperature, using a Jandel multi-height four-point probe with 500-micron tip radius and 1.00 mm spacing (Jandel Engineering Ltd., Linslade, Beds, UK). Conductivity (S/cm) was calculated as the inverse of resistivity. The resistivity of a membrane was the average of 10 measurements at randomly selected locations.

#### 3.1.2. Cyclic Voltammetry

The electrochemical cyclic voltammetry (CV) was performed using the potentiostat/galvanostat/impedance analyzer PalmSens4 (Bioanalytical Systems Inc., West Lafayette, IN, USA). For the three-electrode electrochemical evaluations, the as-prepared PPy membranes were cut into circular specimens of 1 cm in diameter and used as working electrodes, while a Ag/AgCl electrode and Pt mesh (2.0 cm × 2.0 cm) were used as reference and counter electrodes, respectively. The electrolyte was 1.0 M H_2_SO_4_ aqueous solution. The cyclic voltammograms were obtained in a potential window of 0.0–0.8 V at a scan rate of 0.01 V/s.

#### 3.1.3. Galvanostatic Charge–Discharge

Galvanostatic charge–discharge (GCD) test was performed using the same instrument in cyclic voltammetry. The electrochemical system and sample size were also exactly the same. The potential window was 0.0–0.8 V and the current densities were 1, 2, and 4 mA/cm^2^. The areal specific capacitance calculation was performed based on obtained results of 1 mA/cm^2^ of current density.

#### 3.1.4. Electrochemical Impedance Spectroscopy (EIS)

The sample preparation, cell system, and instrument used in EIS characterization were the same as in cyclic voltammetry. Sample characterization was performed at room temperature in the frequency range of 10.0 kHz to 0.1 Hz by imposing 5.0 mV AC sine waves on 0.8 V DC potential with 10 points/decade. The response of the membranes included AC and DC components that were converted to impedance values at different frequencies to build the impedance spectra [15,16]. Data were fitted into equivalent circuits using the fitting program of the EIS Spectrum Analyzer.

### 3.2. Morphological Characterization

The morphology of the samples was characterized using a scanning electron microscope (SEM, PHILIPS/FEI, Quanta 250 FEG SEM, PHILIPS/FEI, Hillsboro, OR, USA) operated at an accelerating voltage of 20 kV. The specimens were coated with gold in a sputter coater (Fison Instruments, Polaron SC500, Uckfield, UK) to reduce the noise in images.

### 3.3. Chemical Characterization

#### 3.3.1. Attenuated Total Internal Reflectance Fourier Transform Infrared Spectroscopy (ATR-FTIR)

To measure the chemical composition, FTIR characterization was performed using the Nicolet Magna-IR 550 spectrophotometer (Nicolet Instrument, Inc., Madison, WI, USA) in attenuated total reflectance (ATR) mode. Specimens were pressed against a hemispherical ATR crystal and scanned 64 times between 500 and 4500 cm^−1^ at a resolution of 4 cm^−1^.

#### 3.3.2. X-ray Photoelectron Spectroscopy (XPS)

The surface chemical composition of the membranes was analyzed with a Perkin Elmer PHI 5600 X-ray photoelectron spectroscope (XPS) (Perkin Elmer, Eden Prairie, MN, USA). The take-off angle of the emitted photoelectrons to be measured was 45° with respect to the normal of sample surface, and the analyzed area was 0.5 cm^2^. The vacuum in the sample chamber was maintained at a pressure of 8 × 10^−9^ torr. Both survey scans and high-resolution scans of C_1s_, O_1s_, and N_1s_ were performed without charge neutralization, and with the standard aluminum X-ray anode. The spectrometer work function was adjusted to give 285.0 eV for the main C_1s_ peak. Curve fittings for high-resolution peaks were determined by means of the least squares minimization procedure employing Gaussian-Lorentzian functions and a Shirley-type background, using the MultiPak software. For each membrane, the presented results correspond to the average value of three tested specimens.

### 3.4. Thermal Stability Study

The thermal stability of the membranes was measured with a thermogravimetric analyzer (TGA2 STAREe, Mettler-Toledo, Schwerzenbach, Switzerland) in the temperature range of 25–600 °C at a heating rate of 20°/min. Data analysis was performed using the STARe software (version 16.10) of the instrument manufacturer. The TGA test was run in duplicate for each membrane.

## 4. Results

### 4.1. Electrical Conductivity

The surface conductivity of the PPy membrane was 1.6 ± 0.2 S/cm and that of the PPy–MO membrane was 2.5 ± 0.2 S/cm. While the difference is not large, the surface conductivity of the PPy–MO membrane is about 56% higher than that of the PPy membrane.

### 4.2. Cyclic Voltammogram

**Figure 2:**
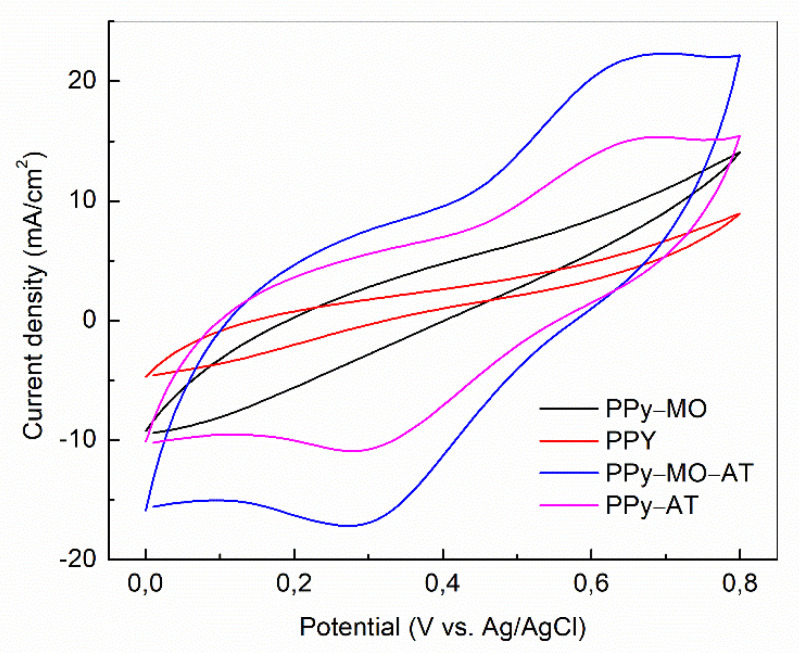
Cyclic voltammograms of different PPy membranes Current density vs. Potential.

The CV curves in Figure 2 are typical for PPy, showing an initial increase in current due to double layer capacitance and then an increase due to redox reaction. What is also evident is that the acid-treated membranes, i.e., PPy–MO–AT and PPy–AT, recorded a steep initial increase and a clear in- and outflux of ions. In a CV test, the area enclosed by the CV curves reflects the amount of charge transfer amount in the system. Consequently, the specific capacitance of the two acid-treated membranes were clearly higher than other two membrane. And the PPY–MO–AT membrane outperformed all other membranes.

### 4.3. GCD Test

**Figure 3:**
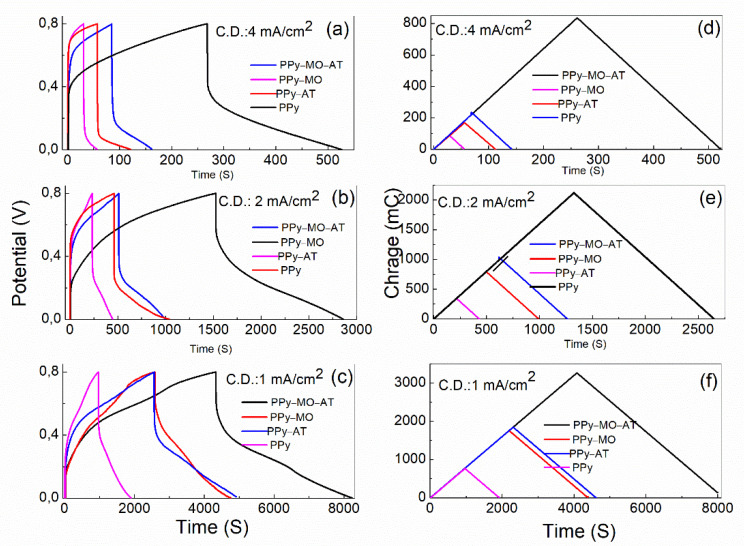
(**a**–**c**): Galvanostatic charge/discharge. (**d**–**f**): Charge curves of the four PPy membranes at current density of 4, 2, and 1 mA/cm^2^, respectively. C.D.: current density.

Figure 3 shows the GCD curves of different PPy membranes that were tested under current densities of 1, 2, and 4 mA/cm^2^ between a potential range of 0.0–0.8 V. It is obvious in Figure 3a–c that discharging time is significantly increased for the PPY–MO–AT, and the charge curves (Figure 3d–f) confirm that the charge stored in the PPY–MO–AT is indeed much higher than that in other three membranes. Among other membranes, the PPY–AT and PPY–MO show a longer discharging time and higher stored charge comparing to the PPY membrane. All curves show a good reversibility.

The areal specific capacitance was calculated based on the below equation [17]:

$$C_{a}=\frac{I\Delta t}{A\Delta V}$$

where *C_a_* is the areal specific capacitance (mF/cm^2^), *I* is the discharge current (mA), Δ*t* the discharge time (second), *A* is the area of working electrode that is 0.78 cm^2^, and Δ*V* is the potential window (*V*) after deduction of IR drop. The calculated data are listed in Table 1. All data, supported by the CV results, confirm that using MO and acidic treatment has significantly increased not only the specific capacitance but also the discharging time. Table 1 also shows that the effects of MO and acid treatment are roughly additive. For example, to subtract the specific capacitance of the PPy membrane from the addition of the PPy–MO and PPy–AT membranes roughly equals the specific capacitance of the PPy–MO–AT.

### 4.4. Electrochemical Impedance

**Figure 4:**
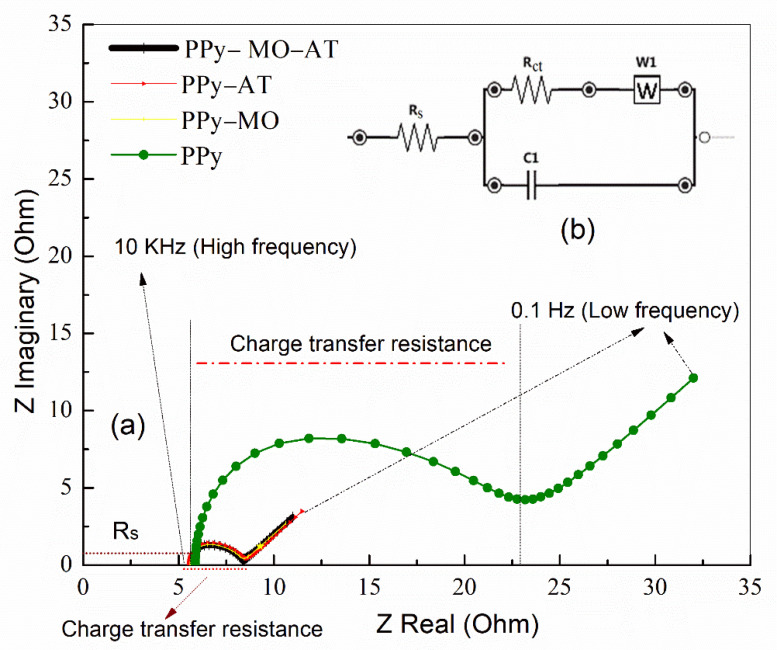
(**a**): Impedance spectra of the 4 PPy membranes in 1 M H_2_SO_4_ at 0.8 V potential and from 0.1 to 10,000 Hz. (**b**): Randle equivalent circuit used to represent the PPy membranes.

The Nyquist plots of the representative membranes are showed in Figure 4. In the Nyquist plot, the linear portion of the curve is referred as the Warburg impedance, which is related to the diffusion-controlled process. The semi-circle portion is related to the double-layer capacitance, the resistance of the electrolyte inside the porous membrane, the internal resistance of the active material (PPy), and the interfacial resistance between the electrolyte and electrode surface [16,18]. The Figure also shows the Randle equivalent circuit in which the solution resistance R_s_ is in series with two parallel branches, of which one branch consists of the Warburg impedance (W) in series with the charge transfer resistance (R_ct_) and the other branch is assigned to the capacitance (C_dl_).

As shown in Figure 4, all three membranes, except the PPy, show similar behaviors. For the PPy membrane, the charge transfer resistance R_ct_ was about 15.6 Ω, which is much higher than that of other three membranes showing the R_ct_ about 2.6 Ω. The Warburg impedance of the PPy membrane, which appeared in the low frequency region and corresponded to diffusion-controlled process, was about three times greater than that of the other membranes, indicating the lack of ions on electrode surface causing an increase in impedance. In term of the double layer capacitance (C_dl_), there was no difference among the four membranes. The numerical values of the components in Randle equivalent circuit are listed in Table 2.

### 4.5. SEM Observations

**Figure 5:**
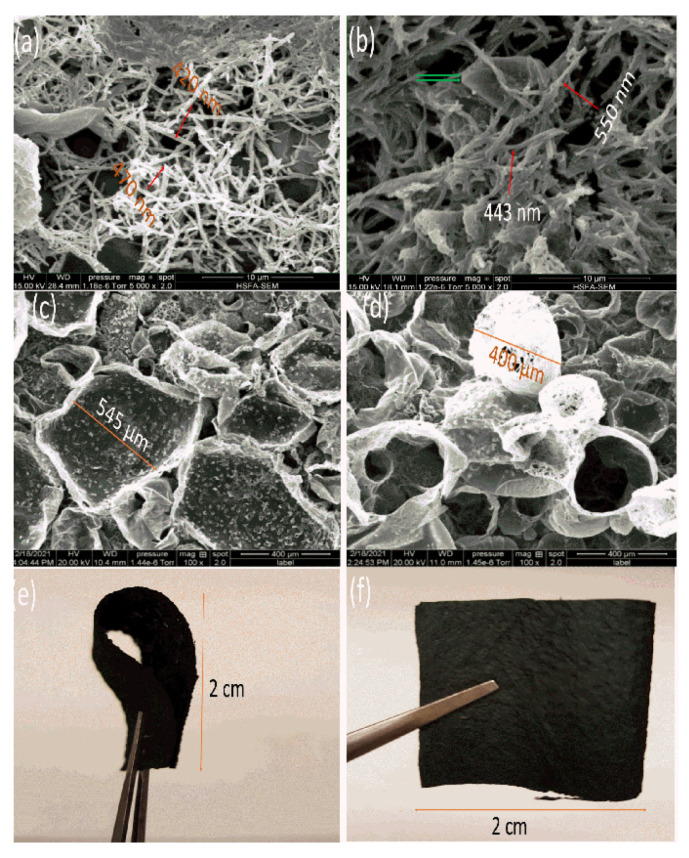
Scanning electron photomicrographs of the PPy (**a**,**c**) and PPy–MO (**b**,**d**) membranes at different magnifications (**a**,**b**: 5000×, **c**,**d**: 100×). The fibrous structures appear on the water-facing surface of the membranes (**a**,**b**) and the bubble-like structures appear on the chloroform-facing surface of the membranes (**c**,**d**). (**e**,**f**): The optical photographs demonstrating the appearance and flexibility of the PPy–MO membrane. Red arrows: PPy fibers. Green arrows: crystals of FeCl_3_/FeCl_2_.

Figure 5 shows the SEM photomicrographs of the PPy–MO and PPy membranes. The chloroform-facing side (Figure 5a and Figure 4b) of both membranes consists of bubbles or collapsed bubbles, showing no significant difference. However, their water-facing side shows a significant difference between the washed (PPy) and non-washed (PPy–MO) membranes. While both membranes are covered by a layer of interconnected fibrous PPy structures (arrow), the diameter of the fibers on the PPy–MO is thicker and many fibers are in micrometer range, which is significantly larger than the nano-sized fibers on the PPy membrane. On the other hand, crystal-like particles (double arrow) are observable on both PPy–MO and PPy membranes and are less numerous on the PPy membrane. MO is known to form nanostructures and to interact with PPy [19], and the fibers became finer after washing; therefore, these thick fibers on PPy–MO are considered the fusion of PPy and MO. The fine fibers on the PPy membrane indicate that most of the MO were washed away. The large particles are considered ferric chloride or ferrous chloride due to their crystalline appearance. The procedure of washing reduced the amount of MO and ferric chloride on the PPy membrane but did not eliminate them completely. Acidic treatment did not affect the membrane morphology and the images are the same.

### 4.6. FTIR Observations

**Figure 6:**
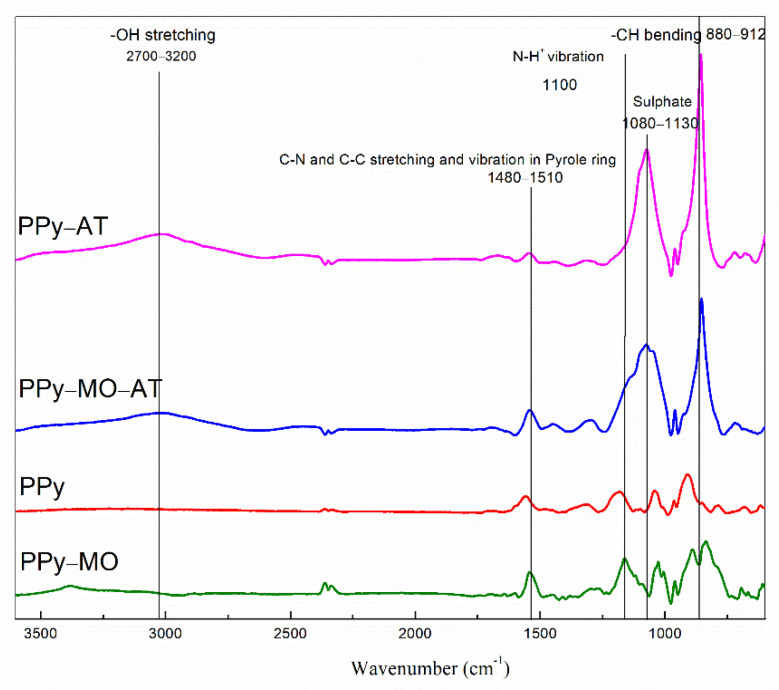
FTIR spectra of PPy membranes.

The FTIR spectra of the membranes are presented in Figure 6. The characteristic bands of PPy show the C-H bending at 880–912 cm^−1^ [20], the C-C and C-N stretching and vibration mode of the pyrrole ring at 1480–1510 cm^−1^ and a broad peak at about 2700–3200 cm^−1^, indicating the presence of OH bonds in carboxylic acid [20,21,22]. The other peaks corresponding to sulfuric acid, MO, and ferric chloride are also indicated in the FTIR spectra [22].

### 4.7. XPS Observations

**Figure 7:**
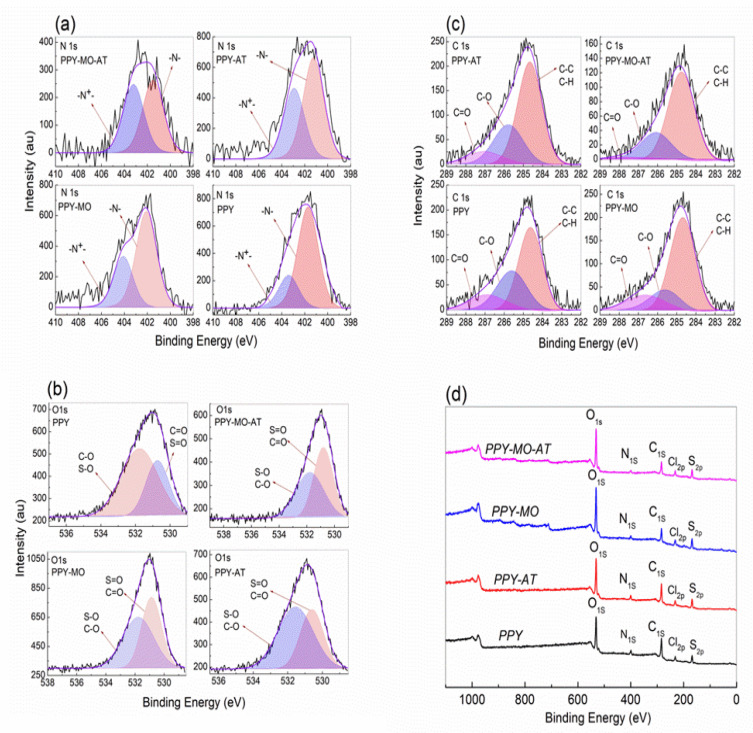
High resolution XPS spectra of N1s (**a**), O1s (**b**), and C1s (**c**) of the different PPy membranes (**d**), and their survey spectra.

Table 3 shows the elemental composition of the four different PPy membranes identified from survey scan (Figure 7d), and the doping ratio defined as the ratio of the oxidized to the neutral nitrogen atoms identified through curve fitting of the N_1s_ spectra (Figure 7a). As one can see that the doping ratio in the PPy–MO membrane is about 1:3 and in the acid treated PPy–MO–AT membrane is 1:2. However, it is only 1:4 in the PPy membrane. In addition, the sulfur content in the PPy membrane is less than that in other membranes. A high oxygen content was found in all membranes, ranging from 33.3% to 48.1%. Chlorine detected in the PPy–MO–AT and PPy–AT membranes was less than 0.1%, and that in the PPy–MO and PPy membranes was 0.4% and 0.1%, respectively. The oxygen may come from MO and the oxidation of PPy. Acid treatment increased S element, reduced C in PPy–AT, and did not affect oxygen content. The high-resolution spectra of C_1s_ and O_1s_ are presented in Figure 7b,c.

### 4.8. TGA Observations

**Figure 8:**
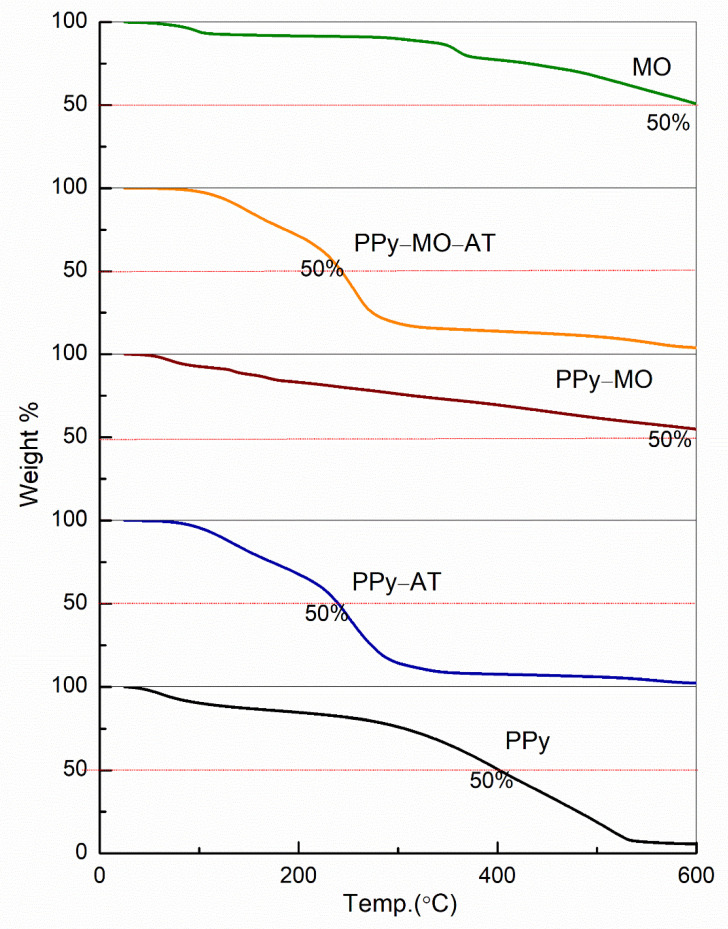
TGA curves of the four PPy membranes and MO.

Figure 8 shows the TGA curves of the four membranes. All membranes had a small weight loss near 100 °C, which was related to moisture in the membrane. The weight loss of the PPy membrane was constant but slow between 100 and 300 °C, and became fast above 300 °C. Acid treatment significantly shifted these two stages of weight loss of the PPy–AT membrane to low temperature side. The impact of acid treatment also applies to the PPy–MO–AT. In fact, the initial weight loss temperatures of the PPy–MO–AT were similar to that of the PPy–AT. However, the residual weight of the PPy–MO–AT at about 300 °C was much higher than that of the PPy–AT, owning to the high thermostability of the MO. The PPY–MO showed almost the same or even better stability with respect to MO, as evidenced by the slightly higher residual at 600 °C. Specifically, both MO and MO (PPY–MO) lost only 50% of their mass at 600 °C. On the other hand, the PPY–AT and PPY–MO–AT lost 50% of their mass at around 240 °C and retained only 10% residual at 332 °C and 512 °C, respectively. The PPy membrane had 10% residual at 600 °C. Table 4 summarizes the TGA data of all membranes including that of the MO.

## 5. Discussion

This work shows that the presence of the micro- and nano-sized fibrous MO and the treatment in 1 M sulfuric acid can significantly increase the specific capacitance of a PPy membrane. Compared with the same membrane, but without MO and acid treatment, the capacitance was increased by about 335% (6417 vs. 2223 mF/cm^2^). In addition, compared with other types of PPy membranes reported in the literature, the method used in this work is very simple and inexpensive. Wang et al. reported a tectorum-like α-Fe_2_O_3_/PPy nanoarrays on a carbon cloth, showing the areal capacitance of 382.4 mF/cm^2^ [23]. In another paper, a PPy/graphene oxide nanocomposite with the areal capacitance of 152 mF/cm^2^ was fabricated [24]. Moreover, Wei et al. fabricated a PPy–cotton electrode by in situ polymerization and achieved the capacitance of 74 mF/cm^2^ [25]. Among these PPy composites, the PPy–MO–AT membrane reported in this work has the highest areal specific capacitance. Based on GCD results, which are also supported by CV data, the specific capacity improvement among the four membranes is in following order:

PPY–MO–AT > PPY–AT > PPY–MO > PPY

Microscopic characterization showed that the PPy–MO and PPy membranes are very similar, presenting micro- and nano-sized fibrous structures on the water-facing side and a bubble-like structure on the chloroform-facing side. The nano-sized fibrous structure is actually made of the PPy nano tubes [13], while the thick fibers on the PPy–MO are MO nanorods and PPy–coated MO nanorods. The only difference between the PPy–MO and the PPy membranes is that most of the MO was washed out from the PPy membrane, turning the PPy–coated MO nanorods into PPy nanotubes [9]. A high nano/micro porosity is known to increase the specific surface area needed for ion transmission and consequently enhances the specific capacitance [9,10]. In this sense, the PPy membrane should have a higher surface area due to the addition of the inner surface of the PPy nanotubes and so a high specific capacitance, which, however, was not the case in this work. Therefore, the answer is not surface area but something else.

**Figure 9:**
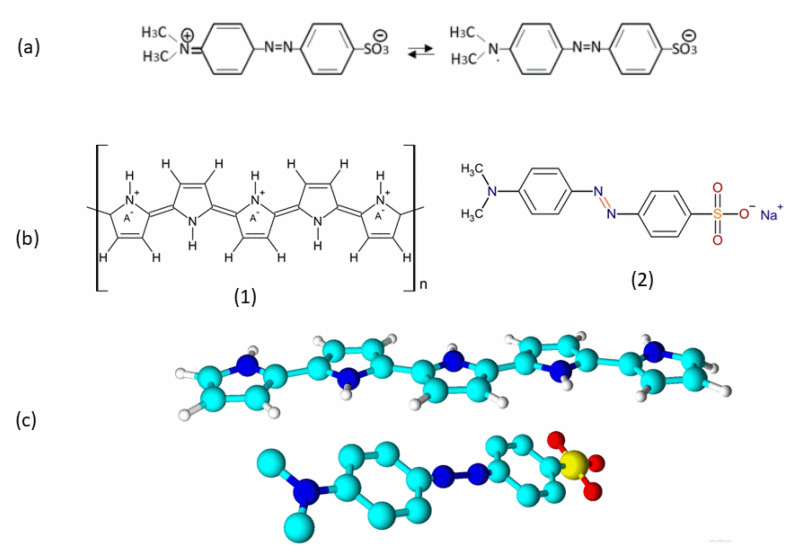
(**a**): Cationic/anionic forms of MO [24]. (**b**): 2D molecular structures of oxidized PPy (1) and MO (2). (**c**): Planar 3D molecular structures of MO and PPy. Software: ACD/ChemSketch (Freeware) 2020.2.1., version C25E41; ACD/3D Viewer (Freeware) 2020.2.1., version D05E41.

MO is an organic semiconductor and its electrical properties are suitable for electronic applications, such as photo galvanic cells [24,25]. MO has a polycrystalline nature with an extended conjugation (Figure 9a) allowing the transfer of delocalized π electrons upon electronic excitation [26,27]. In addition, the 3D molecular structure of MO is flat, favoring stacking with PPy that also has a flat molecular structure if no side reaction at β positions (Figure 9b,c). The impedance, both real and imaginary components, also decrease with the increase in frequency. Therefore, the presence of MO may have improved the molecular regularity of PPy and consequently decreased the impedance of the PPy–MO composite (Table 2).

Another important factor is the treatment with sulfuric acid. MO is known to become bipolar at pH < 3 (Figure 7a), making it a cation/anion exchanger [28]. This increase in molecular polarity should have increased its dielectric constant and consequently capacitance as well.

Similarly, acid treatment to PPy itself can protonate the membrane. It is well-known that protonation in acid can increase PPy conductivity and deprotonation in alkaline condition can inverse it. It is also known that protonation by acid can increase the redox charge transfer in PPy [29]. Indeed, this work also shows that the number of positive nitrogen (N^+^) in PPy chain increased following acid treatment, as revealed by the increased absorption at 1100 cm^−1^ in Figure 6 and by the high doping ratio in Table 2.

The bipolar nature of MO in acid condition also makes it play the role of a counterion. The negatively charged –SO^−3^ is a well-established counterion, such as in the case of polystyrene sulfonate. In this work, the doping ratios of PPy and PPy–MO are 1:4 and 1:3, respectively (Table 2), showing a higher doping ratio when MO was not washed out. Therefore, in the PPy–MO and PPy–MO–AT membranes, there exist two different kinds of dopants, i.e., the free Cl^−^ ions, which come from FeCl_3_, and the –SO^−3^ groups in MO. As mentioned previously, the planar steric conformation of MO (Figure 7c) makes it an excellent structural template for the polymerization of pyrrole. Together with the facts that MO as a semiconductor can transfer electrons by its extended π system, the electronic system of the benzene ring can be excited, and the electrons can be transferred from one molecule to another through the resonance of its unshared electron pair [27]; MO actually contributed largely to the PPy–MO–AT membrane by reducing charge transfer resistivity and increasing specific capacitance.

As can be seen in Figure 4 and Table 4, the presence of MO and the acid treatment dramatically decreased the charge transfer resistivity (R_ct_) from about 15.6 Ω in the PPy membrane to about 2.6 Ω in three other conditions (PPY–MO, PPy–MO–AT and PPy–AT), confirming that the presence of MO and the acid treatment can decrease the inherent resistance of PPy and the contact resistance between electrode (PPy membrane) and electrolyte, and consequently provides more electroactive sites. In addition, the counterions can cross the electrode/electrolyte interface and into the electrode at a higher rate, leading to the high specific capacitance. The Randle circuit shows that the same structure in all membranes (Figure 4b). The model circuit consists of an active electrolyte resistance (R_s_) in series with two parallel branches consisting the double-layer capacitance (C_1_), the faradic reaction component (R_ct_), and the diffusion-controlled process (Warburg impedance or W). The semicircle in Figure 4a found in high frequency region is related to double-layer capacitance and the oblique line found in low frequency region is related to Warburg impedance [18]. Even though the charge transfer mechanism is the same in all membranes, according to the impedance curves (Figure 4), all the circuit components (semicircle and oblique lines) have shifted to smaller values of imaginary and real resistance for the membranes with MO and acid treatment comparing with the washed PPy membrane without acid treatment.

The broad band at above 3000 cm^−1^ observed in the two-acid treated PPy belongs to acidic protonation of the PPy molecules in nitrogen sites (Figure 6). The presence of the sulfuric group (1080–1130 cm^−1^) [29,30] in PPy even after washing shows that MO was strongly bond to PPy in its acidic form (Figure 6). The high percentage of sulfur residue, i.e., 8.6% (Table 2), in the washed membrane suggests that MO molecules formed strong interactions with PPy so that they cannot be completely removed by washing. Such interactions very likely include the polar–polar interactions between the anionic sulfonate groups in MO and the oxidized nitrogen in PPy, leading to a higher doping ratio. However, the high percentage of sulfur, which is higher than that of nitrogen, suggests the presence of other mechanisms. Considering the flat molecular structure of both MO and PPy, aromatic stacking and π−π interactions may have happened as well. It is interesting to notice that the electrical and electrochemical properties of the membrane were improved with the increasing of MO concentration and the acid treatment (Table 1 and Table 2), which are proportional to the doping ratio as well. Indeed, doping ratio increased from 1:4 in PPy to 1:2 in PPy–MO–AT (Table 4), which is unusually high.

Acid treatment decreased the thermostability of the PPy membranes. The first critical degradation temperature for the acid treated PPy was around 200 °C, which was lower than other membranes not treated by acid. Sulfonic acid can degrade PPy through oxidation, leading to a decreased thermostability. Another factor is MO. Indeed, the thermostability of MO is superior to that of PPy (Figure 8). It is obvious that the thermostability of the PPy–MO compared with PPy was increased and it stayed quite stable until 600 °C. The thermostability of the PPy–MO was similar or even better than that of MO alone by showing a slightly higher weight residual at 600 °C. It has been reported that MO can form a complex through stacking with polycations, which is an exothermic enthalpy process at high temperature [31]. This may explain the superior thermostability of the PPy–MO membrane. However, acid treatment can also remove some MO from the polymer structure and consequently reduce the thermostability of the membrane. Material thermostability is an important issue in all energy storage devices mainly due to Joule heating. A higher thermostability means a higher working temperature of the device. Based on TGA data, the thermal resistance of the PPy membranes is in the following order: PPy–MO > PPy > PPy–MO–AT > PPy–AT.

## 6. Conclusions

Based on obtained results and calculated specific capacitance in different current densities, it is concluded that both MO and acidic treatment are in favor of electrochemical performance of the PPy membrane. While using MO or acid treatment separately can have a good effect on specific capacitance, the effects of MO and acid treatment can be superimposed to significantly increase the areal specific capacitance of the PPy membrane. The mechanisms may include enhanced polarization of MO, high doping ratio in PPy, and aromatic interactions between MO and PPy. These three processes may have decreased the intermolecular charge transfer resistivity. The thermostability of the PPy–MO membrane was significantly increased, probably due to the stacking between MO and PPy. Acid treatment was found to decrease membrane thermostability. In general, MO and acid treatment are in favor of areal specific capacitance and electrical conductivity. Together with its lightweight and flexibility, the PPy–MO–AT membrane may be further explored as the energy storage material in a wide range of applications such as supercapacitors in electrical vehicles, flexible power source for wearable electronics, and energy harvesting in regenerative braking.

## Figures and Tables

**Table 1:** Areal specific capacitance calculated at different current densities.

Areal Specific Capacitance (mF/cm^2^)			
Membrane Type	Current Density (mA/cm^2^)		
	1	2	4
PPY	2225.7	1381.2	574.0
PPY–MO	4207.4	3840.6	1242.3
PPY–AT	4630.2	3156.0	1394.1
PPY–MO–AT	6417.3	4903.6	2389.0

**Table 2:** Impedance elements of the different PPy membranes.

PPy Membranes	Impedance Elements			
	R_s_ (Ω)	R_ct_ (Ω)	W (σ)	C (nF)
PPy	5.9	15.7	9	3.9 × 10^5^
PPy–MO	5.6	2.6	2	3.3 × 10^5^
PPy–AT	5.5	2.8	3	1.9 × 10^5^
PPy–MO–AT	5.6	2.6	2	1.9 × 10^5^

**Table 3:** Surface elemental composition (%) and doping ratio of the PPy membranes measured with XPS.

PPy Membranes	C	N	O	S	Cl	Fe	Si	N^+^/N	Doping Ratio
PPY–MO	38.8	5.0	43.5	9.8	0.4	1.1	1.4	0.43	1:3
PPY–MO–AT	36.3	6.0	44.8	10.6	0.1	1.2	1.1	0.95	1:2
PPY	48.6	7.8	34.4	8.6	0.1	<0.1	0.5	0.25	1:4
PPY–AT	43.8	6.6	38.2	10.2	0	0	1.1	0.70	1:2.5

**Table 4:** Thermal degradation temperatures of the PPy membranes measured with TGA.

PPy Membranes	Remaining Weight Percentage				
	75%	50%	30%	20%	10%
PPy–MO	315	600	-	-	-
PPy–MO–AT	187	243	266	292	512
PPy–AT	173	240	267	282	332
PPy	306	401	465	496	523
MO	432	600	-	-	-

## Data Availability

Data are available upon request.
